# First Self

**DOI:** 10.3201/eid1102.AD1102

**Published:** 2005-02

**Authors:** Gerald N. Callahan

**Affiliations:** * Colorado State University, Fort Collins, Colorado, USA

**Keywords:** Immunologic and biologic factors, Infection

Slowly, purposefully, my mother unbuttons her blouse. It is blue with small white flowers, and the tail is tucked firmly into the elastic waistband of her salmon-pink pants. Beginning at the top and moving down, she works carefully at each of the small plastic buttons.

"Mother," I plead with her, "you don't need to do that."

She smiles at me and continues unfastening buttons. Her padded cotton and elastic bra begins to appear. Her breasts swell pallidly above it.

The room is not well lit. The curtains are drawn, as they always are, against the sun. But I can see more of my mother than I wish to. My father, sitting here with me, says nothing. My wife, Gina, and two other women in the room also sit silently as my mother undresses herself.

I smell her perfume as she works at her blouse, her perfume and the lotion she lathers herself with every morning. I see the wrinkles beneath her arms, the flaps of skin at the elbows. She pulls off the blouse and stands before us with it in her right hand.

Her gray hair sprays in every direction. Her back is littered with small brown moles, her skin like ice over an old pond. And her dark eyes, fallen far back in the sockets of her skull, flutter from face to face like moths.

This is not, of course, my mother. My mother would never have bared this much of herself in front of strangers and certainly never in front of her son. My mother was quiet, shy, prudent. And this is, of course, my mother, her face, her hands, her dried out, fungus-ruined feet. But things have changed.

The nondescript, nappy, brown carpet is just as it always has been. The counters are still lined with the detritus of middle-class life. The cheap fan still chops at the air overhead, and the draining board with its plastic dish rack still drips dishwater into the same stainless steel sink. The clock with its three golden balls spools out the hours like kites, just as it always has. But my mother is mad. And the six of us have gathered today to evaluate her for custodial care. Custodial care! It sounds as though we might turn her over to the janitors at the university where I work. As though they might know what to do with her since we don't. Appalled or not, though, we have no more time to twist our tales.

## The Self

I am an immunologist. I have spent my life studying the intricate paths by which we protect ourselves from this infectious world, studying self, non-self, and why the two should never meet. But as a son watching his mother disintegrate, I am cut adrift.

My mother's self, the thing that was her for all these years, the thing I had imagined fixed as flint beneath her bones, has fractured, shattered like a crystal vase dropped on concrete. It is one thing to watch feathers grow from chicken-skin grafts on nude mice, quite another to watch your mother undress herself in front of total strangers.

Merriam Webster says that self is "the entirety of an individual, the realization or embodiment of an abstraction" (*1*). I don't know what that means. Even though it somehow feels right, it seems woefully incomplete and metaphysical. As though no human using ordinary language could truly speak of my mother's disappearance, no matter how concretely and obviously she is disappearing.

Sir Frank Macfarlane Burnett first described the necessity of biological self after watching an ameba ingest and digest another microorganism: "The fact that one is digested, and the other not, demands that in some way or other the living substance of the ameba can distinguish between the chemical structure characteristics of 'self' and any sufficiently different chemical structure as 'non-self'" (*2*). Later in contemplation of an immune response, Burnett added, "The failure of antibody production against autologous cells demands the postulation of an active ability of the reticulo-endothelial cells to recognize 'self' patterns from 'non-self' patterns in organic material taken into their substance" (*3*). "Demands" for after all, even the most primitive of us do not regularly eat ourselves. And even the most complicated of us do not regularly mistake our bodies for infectious enemies and destroy the very thing that sustains us. Burnett's self becomes something substantial, something unique that our appetites and our immune systems ignore while they chew away at the rest of the organic world.

The fact that on the surface these two selves—the self of Merriam Webster and the self of Macfarlane Burnett—seem incommensurate, we probably owe to the Frenchman Rene Descartes.

## The Divided Self

Descartes, a mathematician and philosopher, found himself one day deeply concerned with the reality of things. What could he truly trust? What was rationally and irrefutably real? We all know that we make mistakes at times about what is real—the monster under the bed, the shadow in the closet, purpose. Most of us just shrug it off, but Descartes was not so easily mollified. He secluded himself in a darkened room at the back of his chateau and set out to discover what was demonstrably real, trustworthy, *certain* (*4**,**5*).

Descartes considered what we learn through the senses, the stuff we see, hear, taste, touch, and smell—the physical world that apparently surrounds us. Is any of it truly real, unquestionably real? No. Almost immediately, he realized our senses can fool us. Dreams provide hard evidence of that. While we are in a dream, we become completely absorbed with false reality. Dreams do not announce to us that they are not "real." And many things in the "real" world (mirages, optical illusions, sleights of hand) do not announce to us that they are false.

Descartes, the inventor of analytical geometry, turned to the reality of mathematics, a priori knowledge—knowledge accessible without sensory perception. Because of his deep investment in mathematics, Descartes thought a priori knowledge inviolable, beyond reproach, above suspicion. But as he delved deeper, he realized that some evil genius might have fooled us about mathematics. Mathematics might be nothing more than an elaborate ruse with nothing whatsoever to do with reality (as many of us suspected in grade school). He was forced to abandon mathematics as well as sensory knowledge. Without mathematics and the physical world the only things left to Descartes were his own thoughts. He realized that rationally and philosophically he could not question the reality of the questioner. It simply wouldn't make sense. So his questions proved his own existence, even if he could not establish the existence of anything else. *Cogito ergo sum*.

Had Descartes been a microbiologist, things might have ended differently. But for the mathematician, the world devolved to one man's thoughts. Descartes rested then, in the midst of an absolutely solitary universe. Two types of things existed, the seemingly real but demonstrably untrustworthy physical world (*res extans*), and the truly real world of the mind (*res cogitans*). These were two completely separate worlds. The one outside our heads was full of machines and ghosts, including our own bodies. The one in our thoughts was concrete, real, essential. Reality flourished inside human thought, specifically Descartes' thought. The rest was doubtful. When he was finished, Descartes had scalpeled the self off the body.

The self, he claimed, was something other than the physical world that surrounds us. Selves did not come from the same stuff as trees, and stones, and arms, and legs, and knuckles, and immune systems. Selves came from somewhere else. Self stuff and body stuff were distinct and immiscible.

But almost 400 years later, as I watch my mother fumble with the tails on her blouse, I am little comforted by Rene Descartes.

## The Biological Self

We finally convince my mother to put her blouse back on and button it. It takes her two tries, but she now has each button in its proper hole. I am embarrassed. She seems unabashedly pleased with herself—what remains of it. She smiles again at me. I turn to one of the women seated quietly across the room. I look for forgiveness or some sort of reassurance that my mother's antics haven't ruined this for all of us.

"She will do perfectly," Jennifer says.

"Does she wander at night?" Melissa asks.

Lesch-Nyhan disease was first described in 1964, by two physicians named Michael Lesch and William Nyhan. Two brothers appeared one afternoon in these doctors' clinic. Both boys were manifesting bizarre but identical symptoms. Much about these original cases turned out to be characteristic of most cases of Lesch-Nyhan disease, which affects boys almost exclusively. And the fact that the disease manifested identically in the twin boys suggested that an altered gene was involved.

Lesch-Nyhan disease is caused by a mutant gene on the X chromosome. Women have two X chromosomes, men only one. So problems on an X chromosome are sometimes hidden in women by the normal allele on the alternate X chromosome. But Y chromosomes have almost nothing in common with X chromosomes. So X-linked mutations are almost always apparent in men.

A single mutation in the gene that encodes an enzyme called hypoxanthine-guanine phosphoribosyl transferase, or HGPRT, is responsible for Lesch-Nyhan disease. In its mutant form, the enzyme does not function. HGPRT catalyzes a biochemical process called purine salvage. Purines are used to make DNA and RNA—the stuff of genes and genetic control and transcription. Because of the importance of DNA synthesis, we have more than one way to make purines. We can synthesize purines from scratch or salvage purines from DNA-breakdown products in our blood (*6*). People with Lesch-Nyhan disease cannot salvage purines. These people must rely on the purines they synthesize from scratch as their only source of purines because their purine-salvage pathways do not work. On the surface, that doesn't seem like such a bad thing. Beneath the surface, the effects are horrifying.

One of the manifestations of Lesch-Nyhan disease is unmistakable. Somewhere between three months and four years of age, boys with Lesch-Nyhan disease begin to self-mutilate, and they become very creative at it. These boys chew off their lips, chew their fingers to bloody stubs. If not restrained, they engage in head banging, arm and leg banging, nose and eye gouging—sometimes blinding themselves. And they may, against their overt wishes, attack their care givers, sometimes doing considerable harm to the ones they love most.

A single change in a single gene results in massive change in human behavior, massive change in self-perception, massively altered self. After all, what is more basic to self-perception and self-preservation than the ability to distinguish self from non-self and eat only non-self? Descartes was wrong. Wrong! Because clearly, and dramatically, and horribly *res extans* warps *res cogitans*.

We know of several infectious agents that also alter human and animal behavior. *Toxoplasma gondii* makes rats fond of cat urine. *Wolbachia* changes insects' sexual preferences. Borna-disease virus shows up more often in people with certain behavioral disorders. *Dicrocelium dendriticum*, a parasitic fluke, makes ants fond of heights and more likely to be eaten by cows, *D. dendriticum*'s primary host. *Euhaplorchis californiensis*, another fluke, makes fish swim in shallow waters, where they are more likely to be eaten by birds, this fluke's primary host. And Streptococcus infections seem to predispose some children to obsessive compulsive disorders (*7*). We will probably find many more infectious microorganisms that alter human behavior.

Our selves are not something ethereal, something forged from a separate reality. Our selves are no different from our livers or our hearts. Our selves are just as susceptible to the effects of breeding and infection as any other part of us. So, just as there is biology of reproduction or respiration, there must be biology of self. Who we are is not simply a matter of spirit or story. It is in our genes—those we are born with, and those we acquire. Genes arose and were preserved over eons to protect us, to provide each of us with some specific edge in the struggle for survival and reproduction. Our genes come from a very long line of survivors and reproducers. In these genes is the template for self.

"She does wander at night," I add, wishing I didn't have to. "Twice, that I know of, Dad found her outside the house in Kanab, Utah, making her way toward town. The first time she wasn't even wearing the bottoms of her pajamas. When he stopped her and asked where she was going, she said 'Home. I'm going home.' My father could not make her understand that she was home."

## The Evolution of Self

If selves are born inside genes, then like all things biological, selves must have an evolutionary advantage and an evolutionary history. In the beginning there was RNA (probably), RNA that snapped together spontaneously from the molecules afloat in the primitive seas. Then there was DNA twisted into long chains and wrapped in bits of fat. Later, true cells appeared. Life—bacteria, archaea, prokaryotes, eukaryotes—a most remarkable gift began to unwrap itself. Everything was suddenly possible. And from the outset there were only three rules that governed us: eat, don't get eaten, reproduce as quickly and as often as possible—three rules alone that would account for all who followed.

First-self had walked onto the stage. Pronouns became meaningful. "Us" was no longer sufficient to describe everything, "me" and "you" were necessary now. While sense of self was perhaps a long way off, self was there, that day, swimming in a thin broth of "other."

Bacteria were a major step up from the muck. They were, after all, living, but they suffered from one huge drawback: each of them had only one cell to work with. That meant then, and still means now, that most bacterial cells had to do everything, all the time, all at once. Each cell had to see, hear, touch, taste, and smell. Each cell had to eat and excrete, reproduce and think. Each cell had to make everything that was needed for the survival of the individual. Because of this, bacteria, though remarkable survivors, weren't and aren't much good at anything beyond simple survival—poetry probably baffles them.

One day, all of this began to change. A few cells got together, cemented themselves to one another with some new glue. A protoplasmic hand reached into the void and another hand took hold. The door of opportunity swung wide open. For the first time, individual cells were freed from necessity. No longer did anyone have to be everything for everyone. No longer did anyone face everything alone.

Cellular specialization took the world by storm. Some cells stopped eating and became eyes (or something that would one day become eyes), others ears, others nerves, others muscles—there were no limits. Taste buds, antennae, pincers, intestines, hearts, tails, legs, arms, muscles, bones, livers, lungs, hair, nails, claws, blood, hide, and horn were all within reach.

But almost immediately, everyone saw that cellular specialization, alone, led nowhere. Before multicellularity could be had, selfishness was needed. The first few multicellular creatures probably shared everything with everyone. After all, they had no means to distinguish among themselves. All that I have is yours, not because of altruism, but because I cannot tell you from me. Such largesse defeated the whole purpose of cellular specialization. What benefit is there to eyes, if what I see I share with the blind who surround me? Remember there are rules. *I* come first. *I* am not to be eaten by others. *I* am to eat others. *I* am to reproduce first. The things *I* see are for *me* and *me* alone.

But "I" had no means yet for such distinction. What or who was me and what or who was not? I could not decide. Before I could reach for the stars, I had to reach within and find some way to know myself from others. Without a sense of self we are little more than bacteria—maybe less.

If I am to keep what I have earned, if I alone, am to benefit from my mutations and absorptions, my specializations, my senses, my motility, I must know self from non-self. My eyes must be for myself. My thoughts must be my own. My heart must beat only for me. I must keep all that I can to myself at the expense of non-self, or I have gained nothing.

Selves leave no fossils, so we cannot know for certain how the first colonial (multicellular) organisms came to sense their selves. But *biologically*, *biochemically*, *basically*, they had to know, everything depended on it. And the biology and chemistry of that knowledge were and are the only things between each of us and the rest of us. The evolved self, the self geneticized. A protein marker, perhaps, carried by every cell inside every one of us. A passport to be checked and rechecked at every interaction. A self to be validated over and over.

For a few millennia, that was probably good enough. But life was changing. Microorganisms discovered parasitism. Once inside another's membrane, food cost nothing, life was simple, and reproduction was almost guaranteed. Now, self discrimination was not enough. Once others learned to hide within self, force was needed to maintain boundaries. Now, we needed immunity to keep us whole. Once again, if our own mutations and adaptations were to serve us, the integrity of self was essential. Infectious diseases posed the first great challenge to the biological preeminence of self. Immune systems quickly found ways to detect and destroy non-self.

In the beginning, biological self was probably nothing more than a simple system for recognition of other and recognition of non-self as food. Now self had teeth. Now self rose like a shield to stand between us and those who would destroy us to further their own journey towards reproduction. We became what we served and protected.

## Infection, Immunity, and Self

Over time, the self grew. Like the brain, layers upon layers of self formed inside living things. Like the cerebral cortex, late in evolution, psychological self arose—self-conception, self-perception, self-deception. But still, like the amygdala in the brain, beneath the complicated and sophisticated self beats the heart of a beast, focused only on food, survival, and sex.

Unlike the brain, the layers of self are strewn throughout the body. In between the layers is immunity and infection. And that, it seems, ties it all together. When the psychological self is stressed, the immunologic defense of self falters. Perceived threats—exams, other men and women, public speaking, air travel—stimulate the hypothalamus to produce corticotrophin-releasing hormone (CRH), which stimulates the pituitary gland to secrete adrenocorticotropic hormone (ACTH). ACTH induces the adrenal glands to produce cortisol. Cortisol suppresses the immune system (*8*). The two selves synergized. Furthermore, the consequence of this change in perception of self or environment is, as you might expect, accompanied by considerable increase in susceptibility to infectious diseases (*9**–**13*). How we think about ourselves and our surroundings changes our resistance to disease.

Infection and inflammation cross the bridge between selves in the opposite direction. Every organ of the immune system is innervated. Every spot where an immune response takes place is hardwired to the brain. Immunology and neurology are irreversibly intertwined. Interleukins produced by activated macrophages and T cells act on the adrenals, the hypothalamus, the pituitary, and the brain stem. In response, moods change, libidos drop, self-perception fogs, appetites trail off, and sleep becomes nearly impossible (*8*).

Infection, inflammation, and immunity shape self. Self-perception derails immunity. Cytomegalovirus and *T. gondii* have been implicated in the etiology of schizophrenia (*14**,**15*). People with bipolar disorder are more frequently infected with herpesvirus type 1 than people without bipolar disorder (*16*). Mice born to mothers infected with influenza virus never develop a lust for exploration (*17*). And of course infection by other parasites, bacteria, and viruses can change animal behavior in unexpected and consequential ways. Infection, at least at times, changes our mental perceptions of ourselves and our surroundings.

Infections change our immune perceptions as well. Among more than 3 million U.S. military personnel followed from 1988 to 2000, the strongest predictors of multiple sclerosis were serum levels of immunoglobulin (Ig) G antibodies to Epstein-Barr virus viral capsid antigen or nuclear antigen (*18*). Multiple sclerosis is a remarkable autoimmune disease in which the immune system attacks the nervous system—self vs. self. And viral or bacterial infections have been implicated in the etiologies of rheumatoid arthritis, diabetes mellitus, Crohn's disease, and some types of thyroiditis—all autoimmune diseases. These examples indicate that at least some infections cloud the immunologic border between self and non-self. As our understanding of infection and immunologic self-perception deepens, more examples will likely surface. Further research will elucidate the interdependence between immunologic and psychological disorders and the link between both and infectious diseases.

Animals protected from birth against infections never develop any functional sense of immunologic self (*19*). Human autoimmune diseases, such as diabetes mellitus, show a correlation with schizophrenia (*20*) and other behavioral disorders. Cause and effect relationships remain obscure, but a link may exist between infectious disease and immunologic as well as psychological perception of self. Infection, immunity, and inflammation, like water on old plywood, sometimes split and sometimes cement the layers of self. But together or apart, self is a brick in the bulwark of human biology.

## Last Self

All her life my mother preferred things simple. She liked jam better than jelly. She loved cornbread and blackstrap molasses, white gravy, melons, "Amazing Grace." She hated driving. She grew up poor, truly poor. Maybe poverty burned up the all the fuel she was saving for complexity before she ever found any. Regardless, her tastes never changed. She was always most comfortable with ordinary things.

I remember her simplicity. But by the start of the second year of her custodial existence, no matter how hard I tried, I could no longer remember much of anything else about how she once was. I couldn't recall when her hair might have been brown and combed, her pants not fat with diapers, her smile less vacant.

The craters of her face were gaunt and empty now. Every cold and flu wracked her lungs and sent mucus cascading from her nose, across her mouth, and onto to the knit sweaters she wore against the cold. Places where her self had played across the geography of her skin were abandoned—empty lots overgrown with weeds. With me, hers was the purest and truest indifference. I loved her for that. I would sit by her for hours simply to bathe in the low, warm light of that indifference.

The smell of urine was everywhere. We were without pretense. She told stories, I pretended to listen. Over and over she spun those stories around me as though they might protect me from something I would have to face when she was gone. Layer after layer of her peeled away until all that was left was bare wood. The weathered boards scoured of paint by the raging storm. Underneath, there was fear, often; a fervent sexuality, always; and hunger—the vestiges of flames that first flickered billions of years ago. Life, infection, and her own defenses had stripped her of everything else. Unadorned self.

The last day she spoke to me, she was dressed in red sweat pants and a loose purple jersey top with long sleeves. Her nose ran. When I came into her room, she was lying in bed staring at the ceiling. She rarely spoke complete sentences. She never recognized me. I sat next to her and for several moments said nothing.

The room was split in two by a red blanket hung as a curtain. On the other side of the curtain was another bed. Sometimes Mom had roommates, but for the last week or two, no one had occupied the other bed. Full or empty, the other bed held no interest for Mother. The floor was covered in spattered beige tiles.

I enjoyed these moments, sitting quietly next to her—moments stolen from reality, shielded from certainty. I wasn't an immunologist here, just an old woman's son wishing for things that could never be. I reached out and held her hand, thin now with thickened blue veins and knuckles fat from inflammation. She turned to me.

"Hello," I said.

"Hello," she said with obvious pleasure.

Then she lifted my hand to her lips and kissed my finger tips.

"How are you?" I asked because whether someone is dying, bleeding to death from a severed limb, or just finishing a pastrami sandwich, that question inexplicably comes to my lips.

"Fine," she said and turned back to the ceiling, smiling. "I'm fine."

I wiped her nose.

Today, she wore no lipstick, and the aides had taken her bridge from her mouth. Four of her lower front teeth were missing. Her tongue fell through the opening when she spoke and twisted her words.

"What would you like to do today, Mom?" I asked not really expecting anything.

She looked up at me with eyes deep-brown as mahogany. She pursed her lips beneath her small moustache. And for a moment her eyes moved off to one side as though she actually thought about what I'd asked. Finally she looked back into my eyes and said to me:

"I'm hungry."

"Then let's eat."

I lifted her into her wheel chair and rolled it down the tiled hall to the dining room. She ate Salisbury steak and mashed potatoes, green beans and corn, peach pie with ice cream. And likely she would have eaten even more if anyone had offered it. As she ate, she stared across the top of her fork at the brown plastic tabletop. I watched her chin moving with the food and her eyes as they chewed slowly on nothing. Neither of us spoke. There was really no need.
